# Analysis of Granulomatous Lymphocytic Interstitial Lung Disease Using Two Scoring Systems for Computed Tomography Scans—A Retrospective Cohort Study

**DOI:** 10.3389/fimmu.2020.589148

**Published:** 2020-10-30

**Authors:** Jennifer J. Meerburg, Ieneke J. C. Hartmann, Sigune Goldacker, Ulrich Baumann, Annette Uhlmann, Eleni-Rosalina Andrinopoulou, Mariette P. C. Kemner v/d Corput, Klaus Warnatz, Harm A. W. M. Tiddens

**Affiliations:** ^1^ Department of Paediatric Pulmonology and Allergology, Sophia Children’s Hospital—Erasmus Medical Center, Rotterdam, Netherlands; ^2^ Department of Radiology and Nuclear Medicine, Erasmus Medical Center, Rotterdam, Netherlands; ^3^ Department of Radiology, Maasstad Hospital, Rotterdam, Netherlands; ^4^ Department of Rheumatology and Clinical Immunology, Faculty of Medicine, University of Freiburg, Medical Center—University of Freiburg, Freiburg, Germany; ^5^ Department of Paediatric Pulmonology, Allergy and Neonatology, Hannover Medical School, Hannover, Germany; ^6^ Institute for Immunodeficiency, Center for Chronic Immunodeficiency (CCI), Medical Center - University of Freiburg, Faculty of Medicine, University of Freiburg, Freiburg, Germany; ^7^ Department of Biostatistics, Erasmus Medical Center, Rotterdam, Netherlands; ^8^ Center for Chronic Immunodeficiency (CCI), Faculty of Medicine, University of Freiburg, Medical Center—University of Freiburg, Freiburg, Germany

**Keywords:** computed tomography, interstitial lung disease, common variable immune deficiency (CVID), cohort study (or longitudinal study), airway disease, granuloma, scoring systems

## Abstract

**Background:**

Granulomatous lymphocytic interstitial lung disease (GLILD) is present in about 20% of patients with common variable immunodeficiency disorders (CVID). GLILD is characterized by nodules, reticulation, and ground-glass opacities on CT scans. To date, large cohort studies that include sensitive CT outcome measures are lacking, and severity of structural lung disease remains unknown. The aim of this study was to introduce and compare two scoring methods to phenotype CT scans of GLILD patients.

**Methods:**

Patients were enrolled in the “Study of Interstitial Lung Disease in Primary Antibody Deficiency” (STILPAD) international cohort. Inclusion criteria were diagnosis of both CVID and GLILD, as defined by the treating immunologist and radiologist. Retrospectively collected CT scans were scored systematically with the Baumann and Hartmann methods.

**Results:**

In total, 356 CT scans from 138 patients were included. Cross-sectionally, 95% of patients met a radiological definition of GLILD using both methods. Bronchiectasis was present in 82% of patients. Inter-observer reproducibility (intraclass correlation coefficients) of GLILD and airway disease were 0.84 and 0.69 for the Hartmann method and 0.74 and 0.42 for the Baumann method.

**Conclusions:**

In both the Hartmann and Baumann scoring method, the composite score GLILD was reproducible and therefore might be a valuable outcome measure in future studies. Overall, the reproducibility of the Hartmann method appears to be slightly better than that of the Baumann method. With a systematic analysis, we showed that GLILD patients suffer from extensive lung disease, including airway disease. Further validation of these scoring methods should be performed in a prospective cohort study involving routine collection of standardized CT scans.

**Clinical Trial Registration:**

https://www.drks.de, identifier DRKS00000799.

## Introduction

Common variable immunodeficiency disorders (CVID) are a heterogeneous group of primary antibody deficiency syndromes (1). Clinical diagnosis is based on a decreased level of IgG, IgA, and/or IgM, an impaired immune response to vaccines, and the absence of defined causes for hypogammaglobinaemia (2). CVID result in a broad spectrum of clinical presentations (3). In the early stages of disease, patients often present with recurrent upper and lower respiratory tract infections. Although the use of immunoglobulin replacement therapy can significantly reduce the risk of lower respiratory tract infection in these patients (4), a substantial proportion of patients develop progressive airway disease (5, 6).

**Figure 1:**
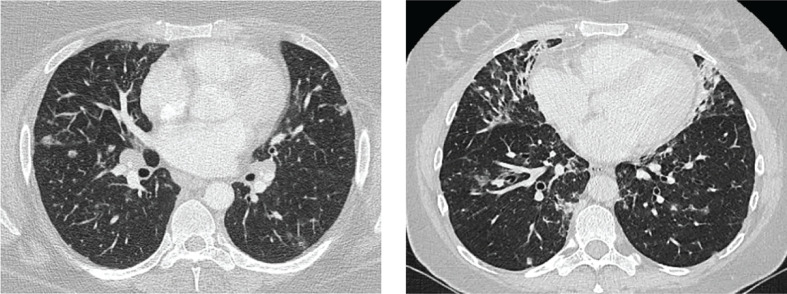
Features of granulomatous lymphocytic interstitial lung disease (GLILD). Images of two study patients. Left: diffuse nodules and lymphadenopathy. Right: combination of diffuse nodules, reticulation and ground-glass opacities. Apart from GLILD features, also signs of airway disease.

In addition, 30%–50% of CVID patients develop non-infectious autoimmune disease, organ inflammation or malignancies. Since adequate immunoglobulin replacement therapy has been introduced, these comorbidities have a larger impact on patient prognosis than the recurrent infections (3, 7). Granulomatous lymphocytic interstitial lung disease (GLILD) belongs to these comorbidities and affects 8%–20% of CVID patients (8, 9). GLILD patients show signs of lymphoproliferative pulmonary disease, including lymphocytic interstitial pneumoniae, follicular bronchiolitis, or lymphoid hyperplasia in combination with granulomas. The diagnosis is made by performing both radiological and histopathological examinations of the lungs (6, 9). Although the pathogenesis of GLILD is not well understood, autoimmune and inflammatory dysregulation and their association with other autoimmune disorders are thought to play a role (5). It was shown that CVID patients with GLILD (n = 13) have a markedly reduced survival rate of 50% compared to patients without GLILD (n = 56) and this finding led to a heightened clinical interest in the GLILD patient group (9). Importantly, this interstitial lung disease can lead to clinical complaints such as reduced exercise tolerance and dyspnoea. Furthermore, GLILD patients have a more complex clinical course, as they tend to have a higher frequency of B-cell lymphoma and autoimmune diseases compared to non-GLILD patients (9, 10). Currently, the gold standard to assess GLILD-related structural lung changes is chest computed tomography (CT). Frequently observed lung abnormalities in GLILD include: ground-glass opacities (GGO), diffuse nodules, lymphadenopathy, diffuse patchy consolidations, and reticulation (9, 11, 12). This is distinct from signs of airway disease, like bronchiectasis, airway wall thickening and trapped air (11, 13–16). Two typical CT images of GLILD patients are shown in **Figure 1**.

Most studies on GLILD-related CT structural lung abnormalities involve retrospectively extracted data from radiologic reports (9, 12, 17–19). However, these reports are generally not well standardized nor quantitative, making it difficult to compare findings.

A more systematic and reproducible approach to quantify these abnormalities is to use standardized CT scoring methods. Outcome measures derived from scoring methods can be used both for research purposes and in clinical follow-up (20). Furthermore, they can be used to phenotype patients for personalized clinical care. Few studies have employed scoring methods to systematically assess chest CT scans of GLILD patients. Van de Ven et al. used a scoring system for paediatric CVID or CVID-like patients (n = 54), which was subsequently applied to adults with CVID (n = 47) (15, 21). Similarly, Gregersen et al. used a simplified scoring method to assess CVID in adults (n = 65) (22). Chase et al. evaluated the efficacy of chemotherapy in seven GLILD patients by assessing CT scans performed before and after treatment (23). A major limitation of these studies is that only a small number of patients with GLILD were included. This warrants the need for larger cohort studies to better understand the radiologic characteristics of GLILD and to optimise methods to quantify disease severity in these patients (6, 9, 24).

From 2012 to 2014, a large international observational study, The STudy of Interstitial Lung Disease in Primary Antibody Deficiency (STILPAD), was initiated by the Centre of Chronic Immunodeficiency at the University Medical Centre Freiburg in Freiberg, Germany. The purpose of STILPAD was to describe the natural course and different treatment responses of GLILD. Fourteen medical centers across three countries retrospectively collected clinical data of 146 GLILD patients, from which all available chest CT scans were analyzed to phenotype pulmonary abnormalities in these patients. The aim of this present study was to assess the radiologic features on retrospectively collected chest CT scans of the STILPAD subjects using and comparing two independent scoring methods developed for CVID patients.

## Methods and Materials

### Study Population

Patients with the clinical diagnosis of GLILD enrolled in STILPAD between 2012 and 2014 were included in this study. Inclusion criteria were as follows: 1) CVID defined by criteria approved by the European Society for Immunodeficiencies and the Pan-American Group for Immunodeficiency (2), 2) age of 18 years and above, and 3) a radiological diagnosis of interstitial lung disease or granuloma on chest CT scan, characterized by the presence of nodules, reticulation, or GGO. This evaluation was performed by the radiologist at each participating medical center.

Given the unresolved discussion whether a histological proof of GLILD is required, a histopathological diagnosis of GLILD was made only in few patients based on the policy of each center, and this was not an inclusion criterion.

### Collection of CT Scans

All available digital CT scans of the STILPAD cohort were collected retrospectively between December 2013 and April 2015. Exclusion criteria for image analysis were as follows: incomplete display of the lung, substantial motion artefacts, pneumothorax, or the absence of a reconstruction series required for lung image analysis. To evaluate the presence and severity of pulmonary abnormalities in GLILD patients, the most recent CT scan of each patient was analyzed. For the assessment of change in disease over time, patients with at least two CT scans were included.

### CT Scan Characteristics

Information on CT parameters, including slice thickness, lung volume during acquisition, volumetric or sequential acquisition, and the reconstruction kernels were noted for each scan.

### CT Scan Analysis

CT scans were scored using two methods developed for scoring CVID CT scans: the Baumann method and the Hartmann method. Key features of these methods are outlined in **Table 1**. Both scoring methods evaluate not only CT changes associated with interstitial lung disease but also airway disease as outlined below.

**Table 1:** Differences between the Baumann and Hartmann scoring methods for common variable immunodeficiency disorders.

	Baumann	Hartmann
Abnormalities scored per	Whole lung	Lobe
Number of values	22	157
Time needed per CT (minutes)	15	30
Origin	Newly designed for CVID as a scoring system for clinical use	Based on the cystic fibrosis-CT scoring method, and designed as a sensitive scoring system for CVID patients for research purposes
Emphysema	Scored together with bullae	Scored as separate entity
Reticulation	The presence and subtype of reticulation (inflammatory, fibrotic, or mixed) are noted	Differentiation between reticulation with or without distortion
Lymphadenopathy	Size of the largest lymph node is measured in mm	Only the presence is scored defined by a short axis diameter ≥ 10 mm
Ground-glass opacities (GGO)	Both the presence and subtype of GGO (inflammatory or fibrotic) are noted	No subtypes of GGO are noted

This table presents the key differences between the Baumann and Hartmann scoring method for computed tomography (CT) scans of patients with common variable immunodeficiency disorders (CVID).

#### Baumann Scoring Method

The Baumann scoring method, shown in **Supplemental Digital Content 1**, was developed by an international interdisciplinary group known as the Chest CT Antibody Deficiency Group. One of its objectives is to standardise the reporting of chest CT findings of patients with antibody deficiencies in a reproducible and clinically applicable manner. The group recently published a report on the distribution of bronchial pathologies in CVID patients in a large international cohort (25). The Baumann method evaluates the presence of 13 different abnormalities without assessing their distribution within specific lobes of the lung. These include: bronchial wall thickening, bronchiectasis (excluding traction bronchiectasis), mucus plugging, atelectasis, nodules, reticulation (“lines”), consolidation, GGO, cysts, emphysema or bullae, linear scars and bands, trapped air, and lymphadenopathy. Briefly, the extent of each abnormality is evaluated by counting the number of affected lung lobes; the lingula being considered as a separate lobe. Furthermore, a score between 0 and 3 denotes the severity of bronchial wall thickening and bronchiectasis. Nodules are divided into three size-based categories and in cases of lymphadenopathy; the size of the largest lymph node is measured. This results in 22 scoring items per CT-scan.

#### Hartmann Scoring Method

The Hartmann scoring method, shown in **Supplemental Digital Content 2**, is derived from the validated cystic fibrosis - CT scoring method, with additional items describing abnormalities typical of immunodeficiency syndromes (26). The Hartmann method evaluates abnormalities in more detail than the Baumann method to detect more subtle changes over time. This method was designed for research purposes and is less suitable for clinical practice due to its extensiveness. In summary, the following abnormalities are assessed: bronchial wall thickening, bronchiectasis (excluding traction bronchiectasis), mucus plugging, atelectasis, nodules, reticulation, consolidation, GGO, bullae and cysts, emphysema, distortion, trapped air, and lymphadenopathy. Unlike the Baumann method, each lobe is scored separately, with the lingula being considered as a separate lobe. The extent and severity of specific abnormalities are scored on a scale of 0 to 3. A total of 26 items are scored per lobe, and lymphadenopathy is only scored once. This results in 157 scoring items per CT scan.

#### Component and Composite Scores

In both methods, individual component scores for bronchiectasis, bronchial wall thickening, mucus plugging, nodules, reticulation and GGO are expressed as a percentage of the maximum score.

Component scores of bronchiectasis and bronchial wall thickening were calculated by multiplying the extent of disease by a factor (multiplier), such that the higher the severity of disease, the higher the multiplier (27, 28). Bronchiectasis severity scores of 1.0, 1.5, 2.0, 2.5, and 3.0 had multipliers of 1.00, 1.25, 1.50, 1.75, and 2.00, respectively. Likewise, bronchial wall thickening scores of 1.0, 2.0, and 3.0 had respective multipliers of 1.00, 1.25, and 1.50.

Besides the component scores for single abnormalities, three composite scores were calculated and expressed as a percentage of the maximum score. The GLILD composite score comprised the combined score of GGO, nodules, and reticulation. The composite score for airway disease consisted of bronchial wall thickening, bronchiectasis and mucus plugging combined. In addition, the total disease composite score was derived from the sum of all scored abnormalities.

In case no signs of GLILD were found with both the Baumann and Hartmann scores, the CT scans were analyzed by a thoracic radiologist (P.C.).

### Observers

The CT scans were scored by two extensively trained observers (a medical doctor and a final year medical student). Observers were trained and certified using standardized chest CT training modules that were developed by a chest radiologist (IH) and the LungAnalysis Core Laboratory. These modules consist of studying a defined list of literature (29), followed by PowerPoint presentations to train used definitions and reference images to be used for scoring. Finally, the observers had to score training batches of CT scans. Furthermore, each observer received one-to-one training sessions with the chest radiologist (IH). For logistical reasons, the scans were divided into two batches (n = 251 and n = 105), based on order of arrival. Each batch was scored by a single observer. To assess inter- and intra-observer reliability each observer re-scored a randomly selected and randomized batch of 25 and 30 CT scans, respectively.

### Statistical Analysis

Patient demographics are reported as mean (standard deviation) and scoring outcome parameters are presented as the median (interquartile range, total ranges).

Agreement within and between observers was determined using the intraclass correlation coefficients (ICCs) of both scoring methods (two-way mixed-effects model, single measurements, studied relationship consistency) (30). ICC ranges are defined as follows: 0–0.39 poor, 0.40–0.59 fair, 0.60–0.74 good, and >0.75 excellent (31).

To investigate changes in disease over time, mixed-effects models (generalized estimating equations) were used for the following CT outcomes of both scoring methods: the component score bronchiectasis and component scores GLILD, and airway disease and total disease scores. Models were adjusted for multiple visits, with p-values <0.05 considered significant.

Square root-transformed Hartmann component scores of bronchiectasis were used, as the assumption of homoscedasticity (constant variance) was not satisfied in the original scale. Likelihood-ratio tests were used to assess whether a nonlinear assumption would better represent the evolution of disease over time.

Statistical analyses were performed using SPSS version 21.0 (SPSS Inc., Chicago, IL) and R version 3.3.1 (https://cran.r-project.org/).

### Ethics Approval

Approval for this study was obtained from the local ethics committee of the University of Freiburg in Freiburg, Germany (IRB: 189/12), and the national ethical review boards of all participating centers. Written informed consent was obtained from all participants prior to inclusion in this study.

## Results

### Study Population

For this CT analysis eight patients from the STILPAD cohort (n = 146) were excluded, because they had no digital CT scans available (n = 7) or the available CT scans did not meet the inclusion criteria (n = 1). Hence, 138 patients were included in this retrospective CT study, of which 88 (64%) females. The mean age at time of inclusion was 45 ( ± 15) years, and mean age of diagnosis was 41 ( ± 15) years.

### Collection of CT Scans

**Figure 2:**
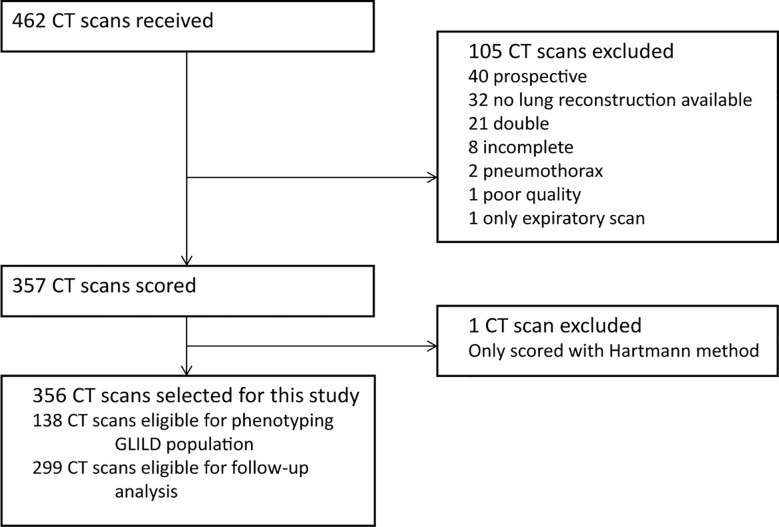
Flowchart CT selection. Flowchart of the in- and exclusion of CT scans. GLILD, granulomatous lymphocytic interstitial lung disease. For phenotyping the GLILD population 138 most recent CT scans were used. A total of 356 CT scans from 138 STILPAD subjects were analyzed and selected for this study. The most recent scan of each patient was used to phenotype the GLILD population. For follow-up analysis, 299 CT scans from 81 patients were analyzed.

A total of 462 CT scans were collected. A flowchart of the CT scan selection process is shown in **Figure 2**. We excluded 105 CT scans as they failed to meet the inclusion criteria and one CT because it was unintentionally scored using only the Hartmann method. Ultimately, the final cohort compromised 356 CT scans from 138 patients.

**Figure 3:**
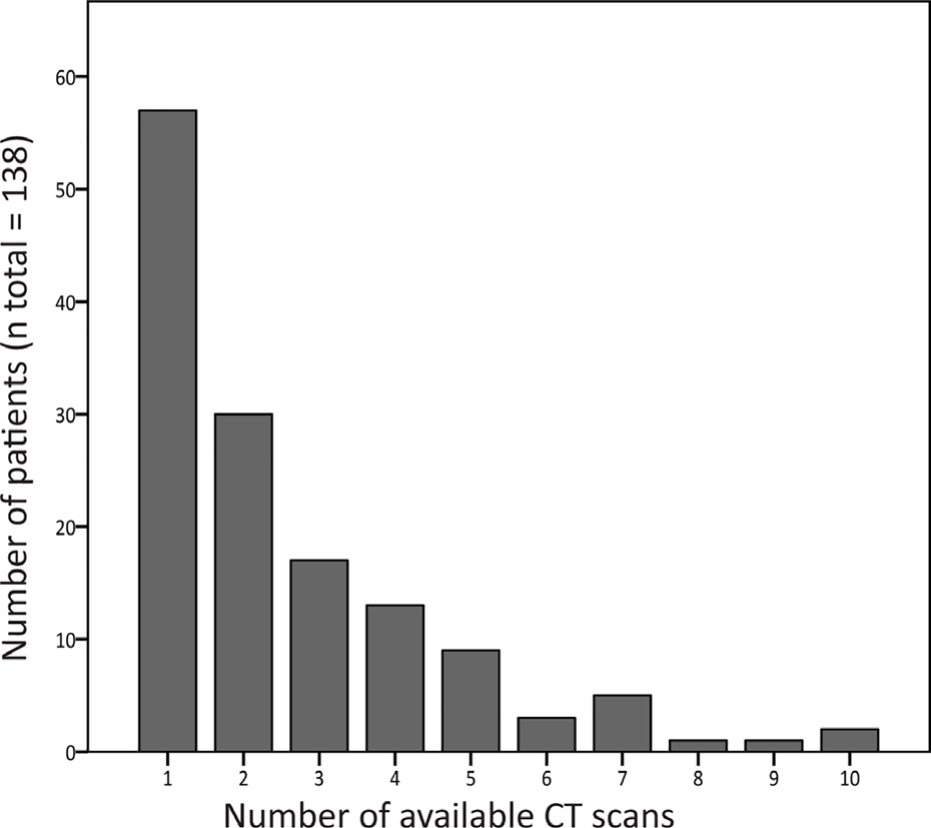
Number of computed tomography (CT) scans available per patient. The number of CT scans that was analyzed per patient is shown in this graph. Of 81 patients, two or more CT scans were collected, and these scans were used for follow-up analysis.

For the longitudinal analysis, 299 scans were collected from 81 patients. **Figure 3** shows the number of CT scans that were analyzed per patient. Median interval (interquartile range, total range) between the CT scans was 12 months (5–24, 0–114).

### CT Scan Characteristics

An overview of the scan characteristics is provided in **Digital Supplement Content 3**. In short: The majority of CT scans (n = 274, 77%) were volumetric. Slice thickness ranged between 0.6 and 8.0 mm, with 267 (75%) of scans having a slice thickness below 3.0 mm. Because only two expiratory CT scans could be collected, trapped air had to be excluded from the analysis.

### CT Scan Analysis of the Most Recent CT

#### Presence of Abnormalities

**Figure 4:**
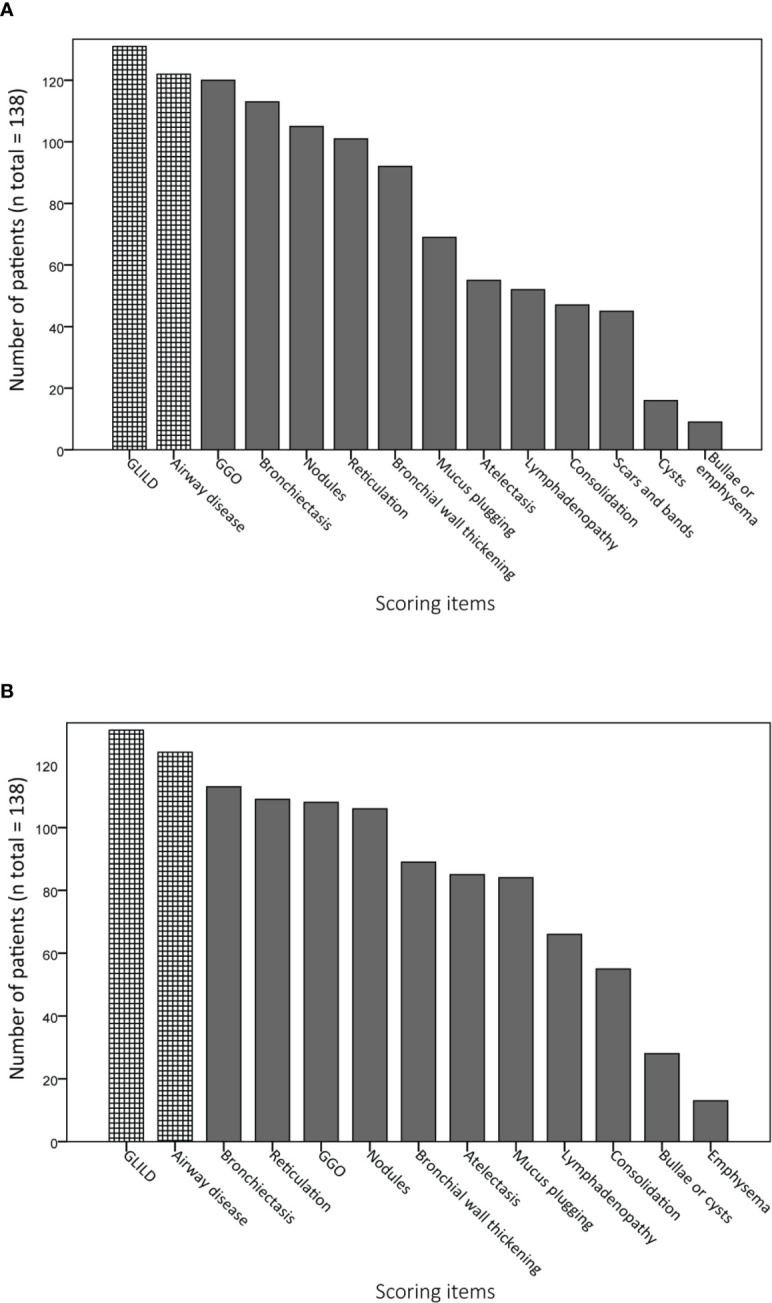
Prevalence of abnormalities on computed tomography (CT) scan. Component and composite scores are sorted based on the number of patients that have a positive score. Granulomatous lymphocytic interstitial lung disease (GLILD) and airway disease are composite scores; GLILD is a combination of component scores for ground-glass opacities (GGO), nodules and reticulation, airway disease is the sum of bronchial wall thickening, bronchiectasis and mucus plugging component scores. **(A)** Scoring items Baumann method. **(B)** Scoring items Hartmann method.

**Figure 5:**
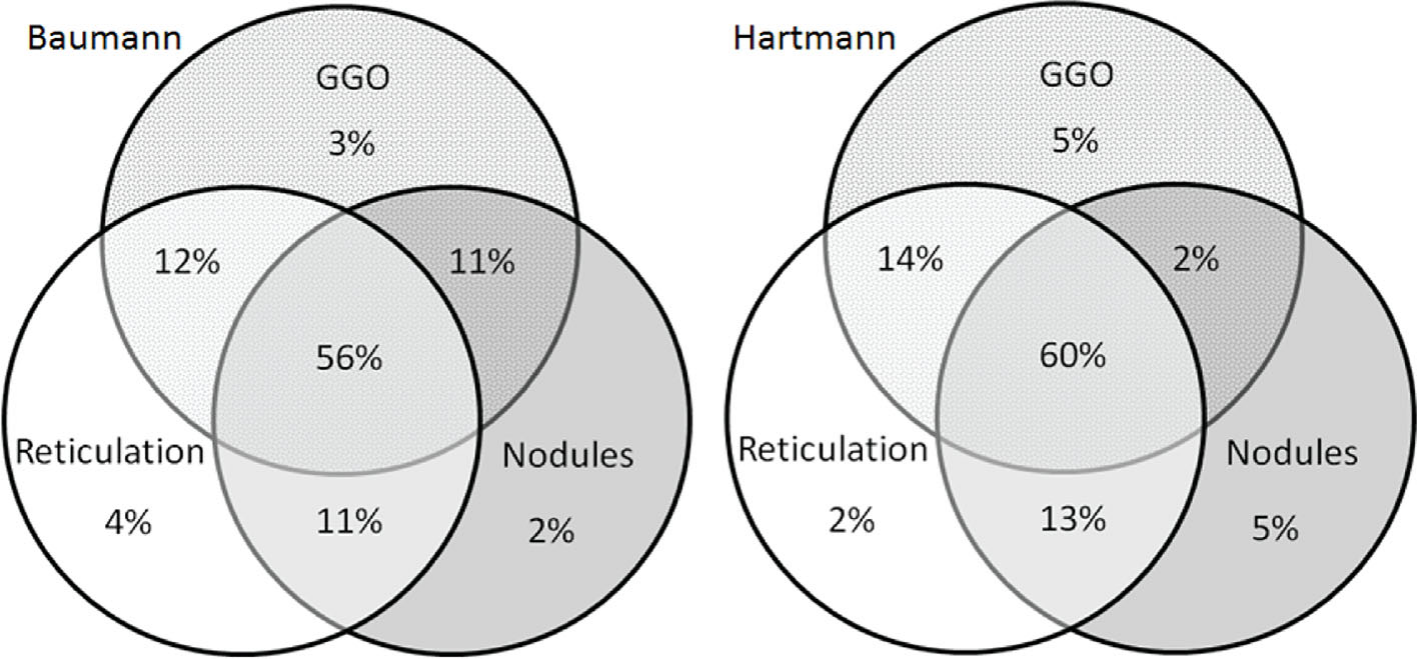
Venn diagrams of features of granulomatous lymphocytic interstitial lung disease (GLILD). Venn diagrams showing the presence of the in the patients method with signs of GLILD on their most recent chest CT scan for both the Baumann (left) and Hartmann (right) (n total = 131). In 56% (Baumann) and 60% (Hartmann) of the 131 patients, all features of GLILD were detected. GGO, ground-glass opacities.

**Figures 4A, B** display the prevalence of component and composite scores of GLILD and airway disease on the most recent CT scan using the Baumann and Hartmann scoring methods. Bronchiectasis was the most common abnormality, with a prevalence of 113 (82%) in all patients for both scoring methods. Other common findings include: bronchial wall thickening, GGO, reticulation and nodules. Signs of GLILD, as calculated by combining the scores of GGO, nodules and/or reticulation, were found on the most recent CT in 131 (95%) of patients for both methods. **Figure 5** demonstrates the relationships between GLILD features. In 56% and 60% of these patients, all features of GLILD were detected with the Baumann and Hartmann method respectively. Signs of GLILD were not detected on the most recent CT scan of five (4%) STILPAD patients in any of the two scoring methods. Of these patients, one patient (1%) had positive GLILD scores on previous scans. The CT scans of the four patients without positive GLILD composite scores on any of their CT scans were re-evaluated by a thoracic radiologist, and signs of GLILD were detected in two of the four patients. Airway disease, defined as bronchiectasis and/or bronchial wall thickening and/or mucus plugging, was present in 122 (88%) (Baumann) and 124 (90%) (Hartmann) of patients. Enlarged lymph nodes were found in 52 (38%) (Baumann) and 70 (51%) (Hartmann) of patients.

#### Severity of Abnormalities

The maximal severity scores for bronchiectasis, bronchial wall thickening and nodules are presented in **Table 2**. Mild bronchiectasis and mild bronchial wall thickening were most frequently observed. In addition, the maximum severity score for bronchial wall thickening was never reached. If nodules were present, the diameter of the largest nodule exceeded the size of 5 mm in 89 (85%) (Baumann) and 87 (83%) (Hartmann) of patients.

**Table 2:** Severity of component scores, bronchiectasis, bronchial wall thickening, and nodules.

Severity of abnormalities	Baumann n (%)	Hartmann n (%)
Bronchiectasis (total)	113 (100)	113 (100)
*Highest score of CT scan*		
Airway >1–<2× vessel	93 (82)	73 (65)
Airway >2–<3× vessel	14 (12)	26 (23)
Airway > 3× vessel	6 (5)	14 (12)
Bronchial wall thickening (total)	92 (100)	89 (100)
*Highest score of CT scan*		
BW > 0.33–<0.5× vessel	85 (92)	75 (84)
BW >0.5–<1× vessel	7 (8)	14 (16)
BW > 1× vessel	0 (0)	0 (0)
**Nodules (total)**	105 (100)	106 (100)
*Highest score of CT scan*		
Largest nodule < 5 mm	16 (15)	19 (18)
Largest nodule >5–<10 mm	46 (44)	43 (41)
Largest nodule >10 mm	43 (41)	44 (42)

Maximal severity scores for the component scores bronchiectasis, bronchial wall thickening and nodules are presented for both methods. Numbers and percentages represent their distribution within the group on the most recent CT scan of patients (n = 138). CT, computed tomography; BW, bronchial wall.

#### Component and Composite Scores

Component scores (bronchiectasis, bronchial wall thickening, mucus plugging, nodules, reticulation, and GGO) and composite scores for airway disease, GLILD, and total disease (comprising all parameters) are shown in **Table 3**. The range between minimum and maximum scores using the Baumann method was wide, particularly for the component scores of bronchiectasis, nodules, GGO, reticulation, and the composite score GLILD which ranged between 0% and 100%. Differences in scores assessed with the Hartmann method were in a lower range compare to the Baumann method, and only the component score for nodules reached a maximum of 100%.

**Table 3:** Component and composite scores as a percentage of the maximum Baumann and Hartmann score.

Component or composite score	Median (%)		Interquartile range (%)		Minimum-maximum (%)	
	Baumann	Hartmann	Baumann	Hartmann	Baumann	Hartmann
Airway disease	17	6	8–30	2–9	0–65	0–44
Bronchiectasis	25	6	8–42	1–11	0–100	0–68
Bronchial wall thickening	22	4	0–44	0–7	0–83	0–49
Mucus plugging	4	6	0–33	0–11	0–67	0–50
GLILD	40	20	20–40	11–31	0–100	0–63
Nodules	22	28	6–56	6–53	0–100	0–100
Reticulation	50	11	0–83	3–17	0–100	0–42
GGO	67	17	33–100	6–33	0–100	0–78
Total disease	21	9	14–28	6–13	0–56	0–32

Component scores of most common abnormalities and the composite scores of airway disease (sum of bronchiectasis, airway wall thickening, and mucus plugging), granulomatous lymphocytic interstitial lung disease (GLILD) [sum of nodules, reticulation and ground-glass opacities (GGO)] and total disease (sum of all component scores) for both Baumann and Hartmann scoring methods are presented as the median, interquartile range, and total range.

### Longitudinal Analysis

**Figure 6:**
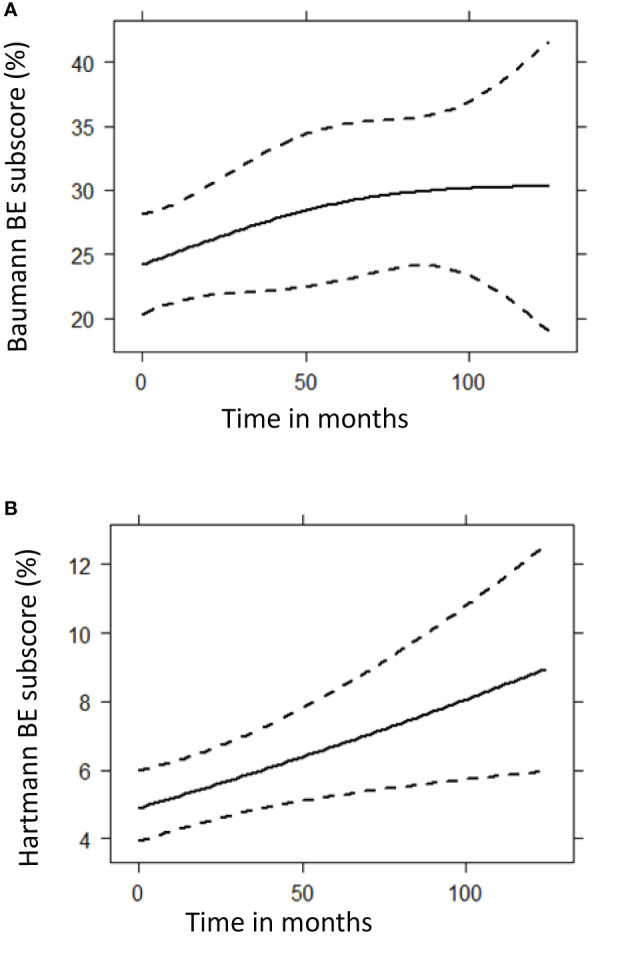
Prediction plots of bronchiectasis (BE) component scores from mixed-effects model analysis. These graphs show the predicted progression in computed tomography BE component scores (%) over time (months) for the Baumann **(A)** and Hartmann **(B)** scoring method, using mixed model analysis. A total of 299 CT scans were used for this follow up analysis. The Baumann BE component score showed no significant change over time (p = 0.1248), while the squared root of Hartmann BE score increased significantly (p = 0.0097). **(A)** Baumann BE component score. **(B)** Hartmann BE component score.

Longitudinal analysis of all follow up scans (n = 299) using generalized estimating equation models showed that the squared root-transformed Hartmann bronchiectasis component score increased significantly over time (p = 0.0097). We found no statistically significant longitudinal change in the Baumann bronchiectasis component score and the Baumann and Hartmann composite scores for GLILD, airway disease, and total disease. Prediction plots of bronchiectasis component scores are presented in **Figure 6**. Complete statistical results of the analysis and prediction plots are displayed in **Supplemental Digital Content 4**.

### Inter- and Intra-Observer Agreement

ICCs of the most common abnormalities are presented in **Table 4**. Both inter- as intra-observer agreement for the Hartmann method was for most items slightly higher than for the Baumann method. Between observers, the Hartmann component scores of reticulation and GGO only had poor inter-observer agreement, while within observers, the agreement for these items varied from poor to excellent. Of the component scores, nodules showed the highest agreement, while bronchial wall thickening and mucus plugging showed only poor to fair agreement. Subtypes of GGO (inflammatory or fibrotic) and reticulation (inflammatory, fibrotic, or mixed), which are exclusive to the Baumann method, showed a poor inter-observer agreement.

**Table 4:** Intraclass correlation coefficients for inter- and intra- observer agreement.

Component and composite scores	Intra-observer 1		Intra-observer 2		Inter-observer	
	Baumann	Hartmann	Baumann	Hartmann	Baumann	Hartmann
GLILD (GGO + NOD + RET)	0.88	0.90	0.85	0.85	0.74	0.84
AD (BE + BWT + MP)	0.48	0.78	0.72	0.76	0.42	0.69
Nodules	0.93	0.90	0.86	0.79	0.78	0.85
Bronchiectasis	0.42	0.63	0.78	0.82	0.53	0.66
Reticulation	0.57	0.66	0.61	0.83	0.47	0.38
Bronchial wall thickening	0.55	0.72	0.45	0.47	0.34	0.49
GGO	0.60	0.38	0.86	0.83	0.44	0.35
Consolidation	0.89	0.33	0.73	0.77	0.55	0.72
Mucus plugging	0.48	0.42	0.52	0.57	0.05	0.38

Inter- and inter-observer agreement expressed as the intraclass coefficient values are presented in this table. Intraclass correlation coefficients were defined as follows: 0–0.39 poor, 0.4–0.59 fair, 0.6–0.74 good, and >0.75 excellent (31). GLILD, Granulomatous lymphocytic interstitial lung disease; GGO, ground-glass opacities; NOD, nodules; RET, reticulation; AD, Airway disease; BE, bronchiectasis; BWT, bronchial wall thickening; MP, mucus plugging.

## Discussion

In this retrospective study, chest CT features of CVID patients with a radiological diagnosis of GLILD were described. A total of 356 CT scans of 138 patients were included and scored using two dedicated CVID scoring systems. A limitation of our study is that histopathological proof of GLILD was rarely available. However it seems that GLILD is not often misdiagnosed in clinical practice: Maglione et al. showed that in 15 of 61 patients in which biopsies were available, diagnosis did not change (16); and Mannina et al. demonstrated that there was no detectable difference between the patients biopsied and not biopsied in regard to the CT morphology or prognosis of the lung function (32). Furthermore, CT patterns compatible with the diagnosis of GLILD were confirmed by the evaluation of the independent readers in this study for all except four participants. Therefore, we consider the effect of lacking biopsy proven GLILD in regard to the goal of this study as minor.

### Phenotyping GLILD Patients

The current pathogenic concept of GLILD comprises mixed T- and B-lymphocytic infiltration of the interstitium of the lungs, partly forming tertiary lymphoid structures next to granulomatous inflammation, follicular bronchiolitis, and reactive lymphoid hyperplasia (6, 33). Typical features of GLILD on CT are patchy GGO, both sharp and unsharp nodules, and reticular lesions varying from fine-lined to course (34). Of the full cohort of 138 included patients, these features were present on their most recent CT scan in 95% of patients, and when also older CT scans were included this was 97% of patients. The two patients, without detectable features of GLILD even after re-evaluation by a thoracic radiologist (P. Ciet), were likely to be misdiagnosed by the radiologists of the participating centers. Overall, this is quite a good result, since reported inter-observer agreement between thoracic radiologists for the diagnosis of general interstitial pneumonia, which has similarities with GLILD, was only 0.52. That of non-thoracic radiologists was even less, namely, 0.48 (35). In the patients with signs of GLILD on their most recent CT, only a small majority exhibited all key features of GLILD. In general, substantial heterogeneity of radiological features was observed in these patients. Enlarged lymph nodes were detected in only 38% of the patients for the Baumann score and in 51% for the Hartmann score. This low prevalence might be explained by the fact that intravenous contrast for better evaluation of lymph nodes was used in only half of the patients. There is no consensus whether contrast medium should be administered in these patients (36). The lower percentage of CTs with lymph nodes for the Baumann score relative to the Hartmann score is probably related to the fact that for this method the exact size in mm of lymph nodes has to be measured which is challenging in the absence of contrast. Other studies report different results: Bates et al. described enlarged lymph nodes in only one out of thirteen GLILD patients (9), while Torigian et al. described enlarged lymph nodes in all five included patients (11).

Although bronchiectasis is not a feature of GLILD, it was the most common CT abnormality, present in 82% of GLILD patients. This result substantially exceeds previously published findings by Torigian (20%), Hartono (35%), Bates (46%), Bouvry (65%), and Mannina (41% diffuse bronchiectasis, 59% focal) (9, 11, 12, 32, 37). Importantly, the patients in some of these studies were younger (9, 11, 32), and in some studies, the interval between time of diagnosis and the CT scan acquisition was shorter (12, 37) Furthermore, the studies by Hartono and Bates did not use scoring methods to analyse the CT scans systematically, which may have led to the underdiagnosis of bronchiectasis. Based on these findings, CVID patients with GLILD have a higher risk of airway disease compared to the risk previously reported for the general CVID cohort (13–16, 25, 38, 39).

### Longitudinal Analysis

Longitudinal follow-up analysis of 299 CT scans from 81 patients showed that only the Hartmann bronchiectasis component scores increased significantly over time. No increase was observed for the composite scores of GLILD, airway disease or total disease. When interpreting the longitudinal data, it is important to consider that we did not correct for any treatment that was given to the patient, and that it is likely that treatment affects the amount of structural lung disease. In a longitudinal study of 54 CVID patients, scores for bronchiectasis and linear and/or irregular opacities were found to significantly decrease while nodules and GGO did not change (14). Conversely, in another study 14 out of 20 CVID patients exhibited worsening of parenchymal changes on their follow up CT scan (13). However, it should be noted that CT scoring was less standardized and statistical analyses were not performed in this study. Maglione et al. presented CVID cases with waxing-and-waning CT features of ILD over time (5).

To study the natural course of disease progression of GLILD, a cohort study involving the routine acquisition of CT scans is required. Importantly, the risk benefit ratio of such a monitoring strategy is warranted as the radiation exposure needed for chest CT is low and taking into account the considerable morbidity and mortality in GLILD patients. Lung volume, CT protocols, and reconstruction kernels should be standardized, in order to improve the diagnostic yield of each CT scan and allow more sensitive monitoring of disease progression (40–42).

### Comparison of Scoring Methods

In this study, two independent CT scoring methods were used to assess GLILD. Baumann scores (**Table 3**) were generally higher, related to the methodology how abnormalities are scored. For example, to compute bronchiectasis component scores for the Baumann method only the most bronchiectatic airways are included. Conversely, to compute bronchiectasis component scores for the Hartmann method also the mean severity of bronchiectasis is included. Consequently, the Baumann method results in higher scores whereas the Hartmann score are in a lower range. Hence, it is not possible to compare the component scores of both methods one-to-one. Longitudinally, the Hartmann method seemed to be more sensitive in assessing bronchiectasis progression over time compared to the Baumann method. The Hartmann method is performed in a lobe-specific manner. Because the Hartmann method provides more precise information about the extent and distribution of lung abnormalities than the Baumann method, this method is more suitable for clinical studies. However, in daily clinical care where time is a limiting factor, the Baumann method might be more feasible to implement.

The Hartmann method also had a slightly higher rate of reproducibility than the Baumann method. The observer agreement for the component score GGO was relatively low for both methods, which might reflect the severe nature of lung disease in GLILD patients: in cases of severe lung disease, the presence and extent of GGO might be harder to assess. Due to the retrospective nature of our study, it is likely that the variable quality of CT scans and reconstruction protocols had a negative impact on the ICCs. Especially the component score reticulation produced low ICCs, which indicates that not all component scores are suitable to monitor GLILD lung disease. Two scoring items exclusive of the Baumann method performed very poor in our study: the subtype of GGO (inflammatory or fibrotic) and subtype of reticulation (inflammatory, fibrotic, or mixed). Thus, these items failed to provide reliable information and to our opinion their relevance is debatable.

However, the component score nodules showed excellent ICCs, and furthermore, the GLILD composite score produced good (Baumann) and excellent (Hartmann) ICCs. A suggestion is to proceed with such scores as main outcomes, while further investigating and improving scoring items with lower reproducibility. Once the relevant changes are agreed upon it will be of interest to transfer the analysis to computer based image analysis in order to render such a scoring method also feasible in regard to time. For this purpose this collection of CT scans will be an excellent resource (43).

## Conclusions

As CT morphology is the one of the major parameters for evaluation during the follow up of GLILD in CVID patients, reliable scoring methods for the longitudinal comparison of interstitial lung changes are required. In this study, we established and evaluated two scoring methods with CT scans of 138 GLILD patients. The composite score for GLILD showed high reproducibility especially according to the Hartmann score, and may become a valuable tool for monitoring disease in longitudinal studies. Once the clinical value of such a score has been demonstrated, automated image analysis systems are needed to optimise the assessment of GLILD and render it suitable for routine diagnostics.

## Data Availability Statement

The datasets, i.e. the CT scores and statistics, presented in this article are not readily available. Proposals may be submitted up to 24 months following article publication. To gain access, requestors will need to sign a data access agreement. After 24 months, the data will be available in the data warehouse of the Erasmus university Rotterdam but without investigator support other than the access to the deposited metadata. Requests to access the datasets should be directed to HT (h.tiddens@erasmusmc.nl) and KW (klaus.warnatz@uniklinik-freiburg.de).

## Ethics Statement

The studies involving human participants were reviewed and approved by the local ethics committee of the University of Freiburg in Freiburg, Germany (IRB: 189/12), and the national ethical review boards of all participating centers. The patients/participants provided their written informed consent to participate in this study.

## Author Contributions

JM: analysis of CT scans, statistical analysis, and first drafts of manuscript. IH: design of Hartmann method and providing training sessions. SG and KW: design and lead of STILPAD. UB: design of Baumann method. AU and MK: collection of CT scans. HT: design of CT study and first drafts of manuscript. E-RA: statistical analysis. All authors contributed to the article and approved the submitted version.

## Funding

This work was supported by Bundesministerium für Bildung und Forschung (BMBF), grant number BMBF 01EO1303. The funding source had no involvement in the study or manuscript preparation.

## Conflict of Interest

SG, AU, and KW report grants from German Federal Ministry for education and research (Grant No. BMBF 01EO1303) during the conduct of this study. KW reports personal fees from Biotest, CSL Behring, LFB Biomedicaments, Baxter, GSK, Pfizer, Novartis Pharma, Roche, Meridian Health Comms, and Octapharma UCB Pharma outside the submitted work, and grants from BMS, CSL Behring, and Biotest outside the submitted work. HT reports grants from Roche, Novartis, CFF, Vertex, Gilead, and Chiesi outside the submitted work, has a patent PRAGMA-CF scoring system issued, and is heading the Erasmus MC-Sophia Children’s Hospital core laboratory Lung Analysis.

The remaining authors declare that the research was conducted in the absence of any commercial or financial relationships that could be construed as a potential conflict of interest.
