# Supplementary material for: Analysis of Granulomatous Lymphocytic Interstitial Lung Disease Using Two Scoring Systems for Computed Tomography Scans—A Retrospective Cohort Study
Source: Front Immunol. 2020 Oct 30;11:589148. doi: 10.3389/fimmu.2020.589148 (PMC7662109; doi:10.3389/fimmu.2020.589148)
Supplement: Supplementary file 1 [file Table_1.docx]

Supplementary Material 1. Scoring items Baumann method

# Table. Scoring items of Baumann method

| **Scoring item** | **Scoring type** | **Meaning score** | **Score range** |
| --- | --- | --- | --- |
| Bronchial wall thickening  Bronchiectasis  Mucus large airways  Mucus small airways  Atelectasis  Nodules <5 mm  Nodules >5 - <10 mm  Nodules >10 mm  Lines  Consolidation  Linear scars and bands  Ground-glass opacities  Cysts  Emphysema or bullae | Extent | Number of lobes | 0-6 |
| Thickest bronchial wall thickening | Severity | 0 = None  1 = BW < 0.5 x V  2 = 0.5 x V < BW < V  3 = BW > V | 0-3 |
| Largest bronchiectasis | Severity | 0 = None  1 = B < 2 x V  2 = 2 x V < B < 3 x V  3 = B > 3 x V | 0-3 |
| Predominant type lines | Pathologic mechanism | 0 = Inflammation  1 = Fibrosis  2 = Mixed type | 0-2 |
| Cause ground-glass opacities | Pathologic mechanism | 0 = Fibrosis  1 = Inflammation | 0-1 |
| Trapped air inspiratory scan  Trapped air expiratory scan  Lymphadenopathy hilar mediastinal | Presence | 0 = No  1 = Yes | 0-1 |
| Lymphadenopathy hilar mediastinal | Size | Size (shortest axis) of largest lymph node in mm | … |

This table presents all scorings items of the Baumann scoring method for computed tomography scans. BW= bronchial wall. V= accompanying vessel. B= bronchial lumen.
