# Supplementary material for: Analysis of Granulomatous Lymphocytic Interstitial Lung Disease Using Two Scoring Systems for Computed Tomography Scans—A Retrospective Cohort Study
Source: Front Immunol. 2020 Oct 30;11:589148. doi: 10.3389/fimmu.2020.589148 (PMC7662109; doi:10.3389/fimmu.2020.589148)
Supplement: Supplementary file 2 [file Table_2.docx]

Supplementary Material 2. Scoring items Hartmann method

# Table. Scoring items of Hartmann method

| **Scorings item per lobe** | **Scoring type** | **Meaning score** | **Score range** |
| --- | --- | --- | --- |
| Bronchiectasis large airways  Bronchiectasis small airways  Bronchial wall thickening large airways  Bronchial wall thickening small airways  Mucus large airways  Mucus small airways  Bullae cysts  Ground-glass opacities  Reticulation without distortion  Reticulation with distortion  Distortion alone  Consolidation  Atelectasis  Emphysema  Trapped air | Extent | 0 = None  1 = >0 - 33%  2 = 33 - 66%  3 = >66% | 0-3 |
| Bronchiectasis large airways  Bronchiectasis small airways  Bronchial wall thickening large airways  Bronchial wall thickening small airways | Extent | Number of segments | 0-5 |
| Largest bronchiectasis  Average bronchiectasis | Severity | 0 = None  1 = B < 2 x V  2 = 2 x V < B < 3 x V  3 = B > 3 x V | 0-3 |
| Bronchial wall thickening | Severity | 0 = None  1 = BW < 0.5 x V  2 = 0.5 x V < BW < V  3 = BW > V | 0-3 |
| Number of nodules | Amount | None  1 = <5 nodules  2 = 5-10 nodules  3 = >10 nodules | 0-3 |
| Contour nodules | Contour | 0 = None  1 = Unsharp  2 = Sharp | 0-2 |
| Largest nodules | Size | None  1 = <0,5 cm  2 = 0,5 - 1,0 cm  3 = >1 cm | 0-3 |
| Pattern trapped air | Pattern | 0 = None  1 = Subsegmental  2 = Segmental | 0-2 |
| Lymphadenopathy hilar mediastinal | Presence | 0 = No  1 = Yes | 0-1 |

This table presents all scorings items of the Hartmann scoring method for computed tomography scans. B= bronchial lumen. V= accompanying vessel. BW= bronchial wall.
