# Supplementary material for: Analysis of Granulomatous Lymphocytic Interstitial Lung Disease Using Two Scoring Systems for Computed Tomography Scans—A Retrospective Cohort Study
Source: Front Immunol. 2020 Oct 30;11:589148. doi: 10.3389/fimmu.2020.589148 (PMC7662109; doi:10.3389/fimmu.2020.589148)
Supplement: Supplementary file 3 [file Table_3.docx]

Supplementary Material 3. Computed tomography scan characteristics

**Table. Computed tomography scan characteristics**

| **CT scan details** | **n (%)** |
| --- | --- |
| Volumetric  Sequential | 275 (77)  81 (23) |
| Slice thickness in mm   - 0.6 - 1 - >1 - 3 - >3 - 5 - >5- 8 | 150 (42)  117 (33)  83 (24)  6 (2) |
| Lung window | 356 (100) |
| Mediastinal window | 335 (94) |
| Contrast given | 175 (49) |
| Expiratory | 2 (0) |

This table presents the computed tomography (CT) scan characteristics of all included scans (n total =356). Data are presented as absolute numbers (n) and percentages.
