# Supplementary material for: Analysis of Granulomatous Lymphocytic Interstitial Lung Disease Using Two Scoring Systems for Computed Tomography Scans—A Retrospective Cohort Study
Source: Front Immunol. 2020 Oct 30;11:589148. doi: 10.3389/fimmu.2020.589148 (PMC7662109; doi:10.3389/fimmu.2020.589148)
Supplement: Supplementary file 4 [file Table_4.docx]

Supplementary Material 4. Prediction plots of composite scores of granulomatous lymphocytic interstitial lung disease (GLILD), airway disease (AD) and total disease from mixed-effects model analysis

# Figure a-f

a. Baumann GLILD prediction plot b. Hartmann GLILD prediction plot

**
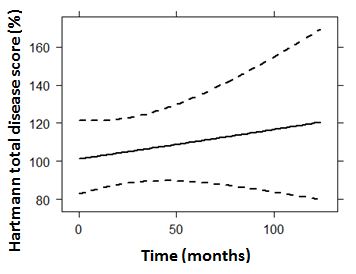
**
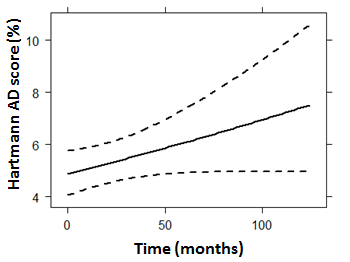

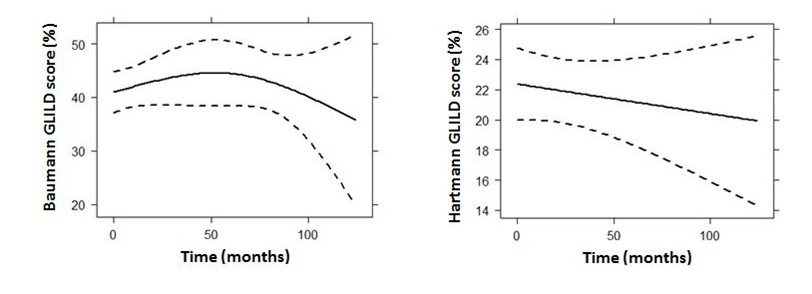


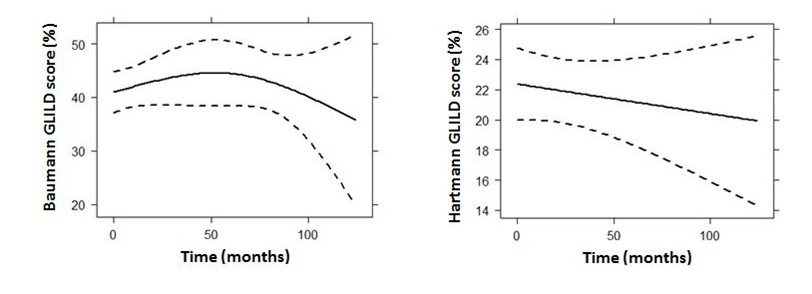


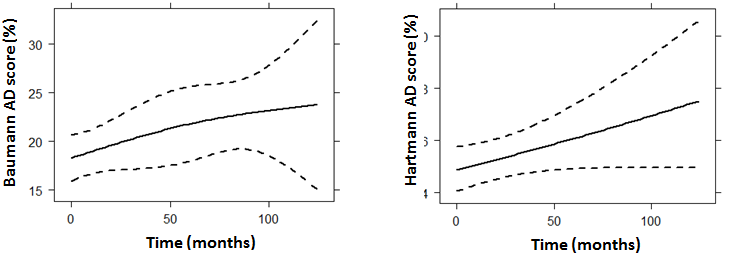
c. Baumann AD prediction plot d. Hartmann AD prediction plot

**
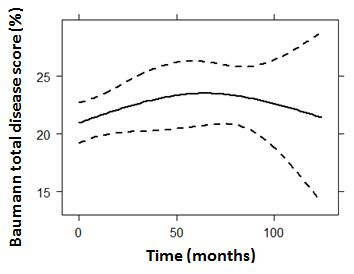
**e. Baumann total disease prediction plot f. Hartmann total disease prediction plot

These graphs show the predicted progression in computed tomography scores (%) over time (months). The composite score GLILD is a combined score of ground-glass opacities, reticulation, and nodules. The composite score airway disease is the sum of bronchiectasis, bronchial wall thickening and mucus plugging scores. The composite score total disease score is derived from the sum of all scored abnormalities. Figure a and b: Baumann and Hartmann GLILD score (p=0.512 and 0.453 respectively). Figure c and d: Baumann and Hartmann AD score (p=0.061 and p=0.073 respectively). Figure e and f: Baumann and Hartmann total disease score (p=0.539 and p=0.171 respectively).
